# Chronic lymphocytic leukemia diagnosis: how many more algorithms and scoring systems do we need?

**DOI:** 10.1016/j.htct.2024.05.007

**Published:** 2024-08-03

**Authors:** Daniel Mazza Matos

**Affiliations:** Flow Cytometry Section, Cell Processing Center (CPC), Center of Hematology and Hemotherapy of Ceará (HEMOCE), Fortaleza, Ceará, Brazil

Dear Editor,

We have read with special interest the article by Ozdemir and colleagues.^1^ The authors propose a new algorithm for the diagnosis of chronic lymphocytic leukemia (CLL) using flow cytometry immunophenotyping. To differentiate CLL from other mature B-cell neoplasms, the algorithm uses the antigens CD5, CD23, CD81, and CD200. As expected, the authors found that their algorithm accurately predicted the diagnosis of CLL, when compared with the classic scoring system by Matutes^2^ and the relatively new proposal by D'Arena.^3^ They concluded that “a new score of four markers (CD5, CD23, CD200 and CD81) is highly effective in distinguishing CLL from other LPDs [Lymphoproliferative disorders].”

Rather than discuss any problem with the article by Ozdemir and colleagues,^1^ I will take this opportunity to examine the limitations and problems that generally affect all CLL diagnostic proposals, whether point-based systems^4^ − where points are awarded according to the dichotomization (positive or negative) of marker expressions – or systems based on formulas^5^^,^^6^ – where the % of positive cells functions as a continuous variable − are used to differentiate CLL from other B-cell chronic lymphoproliferative diseases (B-LPDs).

The scoring systems were originally created with the aim of providing an accurate diagnosis of CLL cases, with the consequent exclusion of other B-LPD, mainly because therapeutic options vary for different mature B-cell neoplasms. Over the years, new markers have been added to the seminal Matutes score,^2^ attempting to improve the accuracy of CLL diagnosis by increasing specificity. The result is that, today, there are just under a dozen diagnostic systems based on flow cytometry available on the ‘market’ for hematologists to choose from. Despite the multiplicity of classification proposals, the fact is that there is a suboptimal agreement between these legions of diagnostic systems, mainly with regard to CD5^+^ LPD that do not have criteria for CLL, such as, for example, some cases of CD5^+^ LPD with three points on the Matures’ score.^4^^,^^7^

This, of course, does not indicate that there is no need for better proposals that could improve our ability to diagnose CLL and, more importantly, to better classify an imprecise group of B-LPD named “borderline lymphoproliferative disorders”: cases of B-LPD in which an accurate diagnosis cannot be established because the immunophenotype of monotypic B-cells does not fit into defined categories such as CLL, mantle cell lymphoma, hairy cell leukemia, Waldenström's macroglobulinemia, marginal zone lymphoma, etc.^8^^,^^9^ However, any future diagnostic system that is based on the same general principles used to date to build current flow cytometry-based classification systems will likely be doomed to suffer from the same limitations as current systems, among which I highlight two: (1) the difficulty in diagnosing some cases of CLL with an atypical immunophenotype and (2) the option of assigning the diagnosis of CLL to CD5-negative B-LPD cases. To overcome these problems, I would like to briefly discuss some points.

With respect to the difficulty in diagnosing CLL cases with an atypical immunophenotype, there is a rationale for the approach of including new immunophenotypic markers in already established scoring systems to increase diagnostic accuracy. Still, it is necessary to be aware that the mere addition of a new marker does not necessarily imply an improvement in diagnostic performance. This occurs because it may happen that the new marker could correlate with others that are already present in the system, and/or its addition could render other markers redundant. In the end, the presence of the new marker could not add diagnostic value to the scoring system.^7^^,^^10^

Notwithstanding, the inclusion of new markers is welcome, even if they initially fail to distinguish between CLL and non-CLL patients. For example, Sorigue and colleagues^11^ recently attempted to use fibromodulin expression by real-time polymerase chain reaction (RT-PCR) to determine whether it could aid in the classification of borderline LPD. Unfortunately, the study did not achieve this goal because borderline LPD patients had fibromodulin expression levels that are consistently intermediate between CLL and non-CLL. Yet, their results suggest that borderline LPD could constitute a distinct nosological entity, perhaps on a biological continuum between CLL and non-CLL LPD.

The second point to be examined is the possibility, using current scoring systems, of assigning the diagnosis of CD5-negative CLL to a patient. I have recently expressed my concerns regarding the real existence of this putative entity.^12^ I will not repeat my arguments here, but I would like to highlight some aspects on this topic.

Tacitly, there are three different perspectives among hematologists and hematopathologists regarding the CD5 expression in CLL. I call them the *flexible vision*, the *inflexible vision*, and the *agnostic vision*. The flexible vision assumes that if the clinical case has a ‘CLL score’ (for example, 4 points in Matutes’ system) then it is CLL (even for CD5-negative cases).^6^ This perspective reveals that one is not aware of the fact that the accuracy of operational criteria (in this case, a given scoring system for CLL) can achieve ‘diagnostic reliability’ but does not confer reality on hypothetical constructs, in this case, the absolutely pure (classic) disease called CLL, for which we would have a gold standard test whereby we could detect ‘variant’ or ‘atypical’ forms of the disease, in this situation a CD5-negative CLL.^12^ This perspective is partially based on ‘consensus’, as in the positions of the European Research Initiative on CLL (ERIC) and the European Society for Clinical Cell Analysis (ESCCA) members.^8^ In effect, less than 5 % of ERIC/ESCCA participants classify CD5 as only ‘suggested’ or ‘recommended’ (as opposed to ‘required’), which nevertheless opens up the possibility of accommodating the diagnosis of CD5-negative CLL. Furthermore, the flexible view is also partially based on the assumption that, in CD5-negative cases, there is a ‘gold standard’ test for the diagnosis of CLL: the presence of proliferation centers (‘pseudofollicles’) in lymph node or bone marrow biopsies. But this is highly dubious. I discussed this point in detail elsewhere, and it appears that there are currently no pathognomonic cytomorphological or histopathological findings for CLL.^12^ Therefore, as a general recommendation, it seems to me that hematologists and hematopathologists should abandon two notions deeply rooted in their conceptions about CLL: (1) that smudge cells, also known as Gumprecht cells (or Gumprecht shadows), in the peripheral blood smears, are useful in the differential diagnosis of CLL^13^ and (2) that proliferation centers are histological structures specific for CLL in the absence of CD5 positivity.^12^ Specifically concerning proliferation centers, this occurs due to the rarity of mature B-cell neoplasms with the immunophenotype identical to CLL in combination with CD5 negativity, which implies that there is not a single large study published to date analyzing the presence of proliferation centers in tissue sections (lymph node and/or bone marrow) in these specific CD5-negative cases.^12^

In turn, the inflexible vision assumes that CLL is necessarily a CD5-positive disease and, consequently, there are no cases of CD5-negative CLL. This view considers that CD5 positivity is a premise for the diagnosis of CLL.^14^ Finally, the agnostic view is close to the inflexible view, but it assumes a statement with a subtle difference: based on *currently* available data, the positivity for the CD5 antigen is a *sine qua non* condition for the diagnosis of CLL. This is my view, and I recently defended it extensively.^12^ Ultimately, based on the published series by Morice,^15^ Rawstron,^16^ and Güell,^7^ the frequency of CD5-negative CLL (if this entity really exists) varies from 0.4 % to 0.9 %, which is far less than that reported by most studies.^12^ In any event, predicated on the absence of a ‘gold standard’ test for CLL, until more evidence is available, namely, a CLL-defining molecular lesion, if one exists, CD5-negative CLL as a true entity may remain unprovable. For cases of B-LPD exhibiting the standard CLL immunophenotype (CD20^dim/neg^, CD23^+^, CD43^+^, CD79b^dim/neg^, CD81^dim^, CD200^+^, FMC7^dim/neg^, ROR1^+^, sIg^dim^) but still distinguished by the absence of CD5, I have previously suggested the provisional name ‘B-cell chronic lymphoproliferative disease with CLL-like phenotype (BCLD-CLL)’.^12^

Taking into account the above considerations, how can new flow cytometry-based classification proposals for B-LPD improve the accuracy of CLL diagnosis, excluding, or at least avoiding, the diagnosis of CLL in CD5-negative cases? One suggestion is to give CD5 a different ‘weight’ in the scoring system. In fact, none of the score-based proposals currently in use give greater ‘weight’ (e.g., assigning a greater number of points) to CD5 within the system. This can be done because, at least intuitively, the diagnostic relevance of all markers (CD5, CD23, CD43, etc.) is not similar and because the diagnostic value of some variables may not reside in their performance but rather in them being a *sine qua non* condition for a specific diagnosis (for a more comprehensive discussion on these points, I refer the reader to the recently published articles by Güell^7^ and Matos^12^).

In conclusion and answering the title question of this article: certainly, novel diagnostic systems and algorithms are desirable, although, in the future, it is not expected that these classification proposals should bring ‘more of the same’, but rather that, either with the inclusion of new markers and/or with the attribution of greater value to CD5, they can refine the diagnosis of CLL and can better separate it from so-called borderline LPD. In the meantime, the search for a gold standard test for CLL is a priority.^12^

## Conflicts of interest

The author reports no conflict of interest.
